# Outcomes of the Internal Medicine Supplemental Application: Preliminary Data on the 2022–2023 Match

**DOI:** 10.7759/cureus.52305

**Published:** 2024-01-15

**Authors:** Elizabeth S Szumel, Holly O Dockery, Seth M Alexander, Debra Bynum, Saumil Chudgar, Donna Williams, Anna Symmes, Katherine Gill

**Affiliations:** 1 Internal Medicine, University of North Carolina at Chapel Hill School of Medicine, Chapel Hill, USA; 2 Internal Medicine, Duke University School of Medicine, Durham, USA; 3 Internal Medicine, Wake Forest School of Medicine, Winston-Salem, USA; 4 Internal Medicine, East Carolina University Brody School of Medicine, Greenville, USA

**Keywords:** national residency match program (nrmp) match, electronic residency application services (eras), graduate medical education, supplemental application, residency selection

## Abstract

Introduction: Preference signaling (program signals and geographic preference divisions) was introduced as a component of the supplemental application for internal medicine applicants applying to programs within the United States (USA) during the 2021-22 cycle. These signals were intended to address application inflation by allowing applicants to express interest in and increase their likelihood of receiving interviews from their top programs. There is little published data, however, to describe the impact of preference signaling on the likelihood of receiving interviews from a program. This study thus sought to analyze, in a small subset of US applicants, whether preference signals were associated with a higher likelihood of obtaining a residency interview.

Methods: A survey was distributed in March 2023 to US MD seniors from the four allopathic medical schools in North Carolina who applied to categorical internal medicine residency programs during the 2022-23 application cycle. The survey was developed by the research team to provide respondents with the opportunity to report data from the electronic residency application service (ERAS) application and provide data on interviews received, actions taken throughout the application season, and outcomes of the National Residency Match Program (NRMP) using a combination of free response and multiple choice questions.

Results: Forty-seven out of a total of 85 contacted (55%) applicants completed some or all of the survey. Of those who completed the entirety of the survey, 39 (82.98%) completed the supplemental portion of the application and the available preference signaling. Applicants in this study were 2.95 (Odds ratio, 95% confidence interval [CI] 2.20 - 3.97, p<0.01) times as likely to receive an interview invitation from a program if they used a program signal. Applicants were 1.75 (odds ratio, 95% CI 1.38 - 2.21, p<0.01) times as likely to receive an interview invitation from a program in an indicated geographic preference division. Forty-seven percent (95% CI 31 - 64%) matched to a program they had sent a program signal to, and 97% (95% CI 78 - 100%) matched to a program in an indicated geographic preference division.

Conclusions: The program signals and geographic preference division components of the supplemental application increased the likelihood of receiving an interview invitation but did not have a clear impact on match outcomes. Further research with larger sample sizes will be necessary to determine how these signals actually modify the outcomes of the NRMP.

## Introduction

With over 50,000 medical students vying for only 40,000 residency positions within the USA, it is increasingly challenging for medical students to distinguish themselves from residency programs and for selection committees to identify applicants genuinely interested in their programs. The establishment of the Electronic Residency Application Service (ERAS) in 1996 allowed applicants to apply to programs electronically, easing the logistical burden of application submission, but also incentivizing submission of more applications and imposing a financial burden on applicants. The transition to virtual interviews in 2020 made it less expensive and logistically easier to attend interviews, but potentially further incentivizing over-application. The change to a pass-or-fail United States Medical Licensing Examination (USMLE) Step 1, a growing number of schools with a pass/fail clinical grading systems, and the dissolution of Alpha Omega Alpha (AOA) and other honor society chapters at many institutions have led to a loss of metrics many residency programs have historically used to stratify applicants [1,2]. As a result of these and other changes, applicants submit more applications; the average US medical student applying to internal medicine (IM) residency through the ERAS applied to 41.3 programs in 2022, a 128% increase from 32.1 applications in 2018 [3].

This increase in applications creates a “prisoner’s dilemma” for applicants; as individuals, applicants would benefit from applying to more programs. However, when many applicants apply this logic and choose to apply to numerous programs, then the collective group of applicants suffers as there are more applications for the same number of residency spots [4]. Top applicants hold a disproportionate number of interview slots; in 2016, the top 12% of IM applicants held 50% of available interview spots, preventing less competitive but well-qualified applicants from being considered by suitable programs [5]. There is a growing concern that the virtual interview process increased this behavior. IM residency programs are also burdened by increasing numbers of applications, receiving an average of 2906 applications in 2021, an increase of 130% from 2018 [6,7]. Application inflation has required many programs to alter recruitment strategies (e.g., adding interview days) and hinders programs’ ability to conduct a holistic review of each application without imposing significant costs [2,8].

Application inflation has highlighted the need to improve the residency application process. To “help programs better identify applicants who are genuinely interested in their program, and whose interests and experience align well with the program’s setting, mission, and goals,” the American Association of Medical Colleges (AAMC) introduced the supplemental ERAS application in 2021. To date, the supplemental application has been considered optional for applicants, individual programs, and entire medical specialties. However, 64% of categorical IM residency programs included supplemental application data in their application review process during the 2021-22 cycle [9]. For the 2023-24 cycle, the supplemental application will be incorporated into the primary MyERAS application, and there will be no separate supplemental application [10].

The supplemental application for the 2022-23 application cycle, completed in addition to the traditional ERAS application, was composed of three sections, in which applicants describe meaningful past experiences, indicate their geographic preferences, and signal their interest in up to seven preferred programs. While the “meaningful experiences” section is meant to expand upon work, volunteer, and research experiences already described in the MyERAS application, the geographic preferences and program signals sections allow applicants to convey new information. While all programs were able to see an applicant’s geographic preferences, only signaled programs could see that they had been signaled. There is limited data to quantify how the use of geographic preference divisions or program signals may impact the distribution of interview invitations, especially within the field of IM. A majority of IM program directors surveyed reported that geographic preferences and program signals helped identify applicants who otherwise might have been overlooked [9].

To date, there has been no analysis of how signals affected the likelihood of an interview offer from IM programs. However, evidence from the 2021 United States otolaryngology match, where signaling was initially piloted, showed that applicants were significantly more likely to receive an interview from a signaled program than from a non-signaled program, regardless of applicant competitiveness [11]. A computer simulation of the otolaryngology match demonstrated that preference signaling would benefit applicants with a strong application, with minimal harm to traditionally strong applications who currently hold the majority of interviews [12]. However, the effect of signaling likely depends on the exact number of signals communicated [13]. There is some concern that allowing preference signaling could paradoxically exacerbate application inflation; if applicants fear that the lack of a signal indicates no interest, they may increase the number of applications sent to non-signaled programs [4].

There is currently insufficient evidence to advise medical students or residency programs on the impact of the geographical preference divisions and program signals within IM. The objective of this study is thus to ascertain the association between the use of the supplemental application and interview invitations for categorical IM applicants during the 2022-23 cycle and whether this association varies with other proxy measures of the strength of a student’s application. As the totality of the data of interest is not collected by any entity, a survey was used to solicit applicant-centered information and collate information into one dataset.

## Materials and methods

To assess the effect of the supplemental application on interview invitations, a detailed survey that elucidated application, interview, and match data was distributed to all 2022-2023 US MD-senior applicants in North Carolina applying to categorical IM programs, encompassing four US MD medical schools. One program was approached regarding US DO-senior applicants, but they declined to participate. Focusing on North Carolina enabled a complete survey of a singular geographic region with both public and private MD institutions, minimizing selection bias.

Survey design and outcomes

The survey was developed by a member of the research team (Szumel) and reviewed by six graduate and undergraduate medical educators and multiple medical students for face validity before distribution. The language of the survey mirrored the language used by ERAS and the National Residency Match Program (NRMP). However, time constraints and lack of an adequate population on which to obtain pilot data from precluded further formal validation of the survey questions.

The primary outcomes of interest were the impact of program signals and indications of geographic preference on the likelihood of receiving an interview invitation. To assess the effects of program signaling and other data from the secondary application, the survey was intentionally designed to capture data from the applicant's ERAS application, the interview process, and the NRMP outcomes. Regarding the primary outcomes, as an example, participants were asked to report the number of programs they signaled and the number of interview invitations they received from signaled and non-signaled programs. Participants similarly reported the specific geographic preference regions they selected and the number of interview invitations received in both preference and non-preference regions.

A secondary outcome was to investigate the relationship between preference signaling and outcomes of the NRMP residency placements. In order to evaluate this association, participants were asked where they matched on their rank list and if they matched at a program they signaled. The survey also assessed other factors reported on an applicant's ERAS application or reasonably reported through the interview process that potentially affect match outcomes, including application to multiple specialties, participation in the Couples Match, number of extracurricular experiences included in the application, communication with programs before and following interviews, and membership in national honor societies. A complete copy of the survey is provided as an online appendix to this article.

Distribution and data analysis

Medical educators from each of the surveyed programs distributed the survey via email to all fourth-year medical students who had applied to IM residency programs. The survey was sent the week following the release of the 2023 NRMP (the Match) results and a week later via a confidential Qualtrics XM link (Qualtrics, Provost, UT). Potential participants had a total of three weeks from the first notification to respond to the survey. Participants were entered to win one of four small financial incentives for their participation.

Descriptive and comparative statistical analyses of response data were completed using STATA BE Version 17.0 (StataCorp LLC, College Station, TX). The alpha level of significance was set at the standard 0.05 significance level for all comparative statistical analyses. Odds ratios (AD/BC) were calculated based on the total number of programs signaled at which an interview offer (A) was received or not (C) compared to the total number of programs that were not signaled at which an interview offer (B) was received or not (D). Similarly, odds ratios were calculated for programs within or outside of a geographic preference region at which interviews were received or not.

This study was reviewed and approved by the Institutional Review Board of the University of North Carolina at Chapel Hill. It was determined to be exempt from federal human subjects research regulations and informed consent was waived (IRB Study 22-2953).

## Results

The survey was distributed to the 85 US MD seniors who applied in categorical IM from four medical schools in the State of North Carolina. Of those, 47 (55%) applicants completed at least some portion of the survey; partial responses were included in the data analysis. Basic demographic information from respondents is included in Table 1.

**Table 1:** Demographic summary based on survey responses. Race and ethnicity categories exceed 100% of respondents (*) as some respondents may have identified with more than one ethnic background.

Demographic	Category	N (%)
Gender Identity	Cis-Female	19 (52.78%)
	Cis-Male	17 (47.22%)
Institution Type	Publicly Funded	25 (53.19%)
	Privately Funded	22 (46.81%)
Age Range	<26 years of age	1 (2.78%)
	26 - 30	30 (83.33%)
	31 - 35	4 (11.11%)
	36 - 40	1 (2.78%)
Race and Ethnicity*	White/Caucasian	21 (60.00%)
	Asian/Pacific Islander	10 (28.57%)
	Black or African American	4 (11.43%)
	Hispanic	2 (5.71%)

Of the respondents, all but three took the USMLE Step 1 as a numerically scored exam with an average score of 242.39 (N=33, Standard Deviation [SD]=13.90, Range 208 - 273). All respondents reported having passed the USMLE Step 1 on their first attempt. The average USMLE Step 2 score was 254.00 (N=36, SD=11.12, Range 228 - 275). Respondents indicated a median of seven volunteer (Interquartile Range [IQR] 4 - 9), three work (IQR 2 - 5), and four research (IQR 3 - 5) experiences, as well as a median of three publications (IQR 2 - 10+).

Applicants applied to an average of 34.62 programs (N=47, SD 18.35, Range 1 - 100); once accounting for programs that applicants withdrew from before interviews were offered, the average was still 34.06 programs (N=47, SD 18.37, Range 1 - 100). Applicants received an average of 16.17 interview offers (N=36, SD=5.81, Range 1 - 28), receiving interviews at approximately 46% (N=36, 95% CI 37.59 - 54.65%) of programs they applied to.

**Figure 1:**
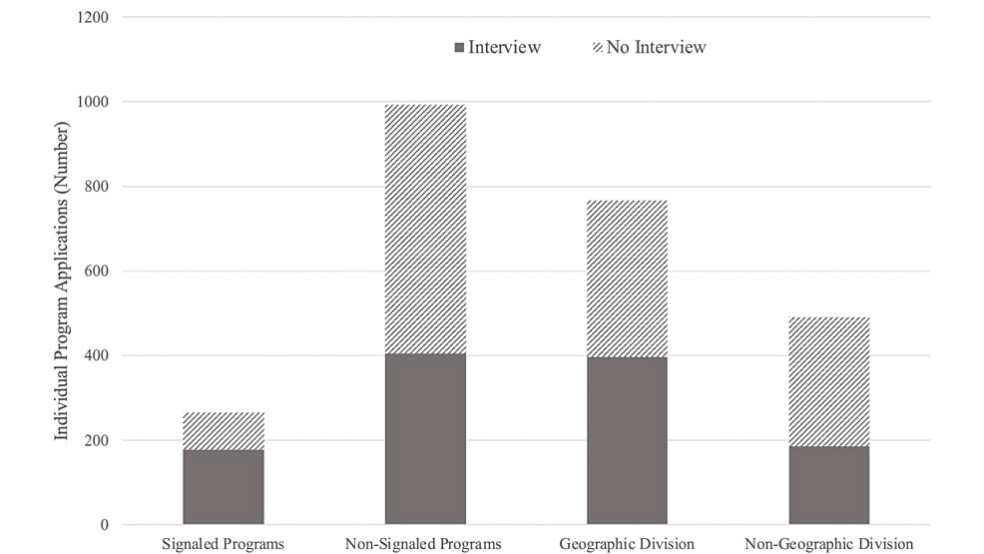
Breakdown of the total number of programs applied to (N=1259) by both program signals and geographic preference divisions and whether each application resulted in an interview offer or not.

Of the respondents, 39 (82.98%) participated in the supplemental application. All respondents (N=38, 100%) who utilized program signals signaled interest in the maximum allowed seven programs, and they indicated an average of 2.26 (N=39, SD=1.16, Range 0 - 3) geographic preference divisions. Approximately 73.40% (95% CI 65.47 - 81.34%) of applications filed were to programs within an applicant’s geographic preference divisions. The odds ratio for obtaining an interview at a program to which the applicant signaled interest was 2.95 (95% CI 2.20 - 3.97, p<0.01). The odds ratio for obtaining an interview at a program in an area indicated as a geographic preference division, regardless of whether a signal was also sent, was 1.75 (95% CI 1.38 - 2.21, p<0.01). A summary of the breakdown of applications and interview offers by program signals and geographic preference divisions can be seen in Figure 1.

**Figure 2:**
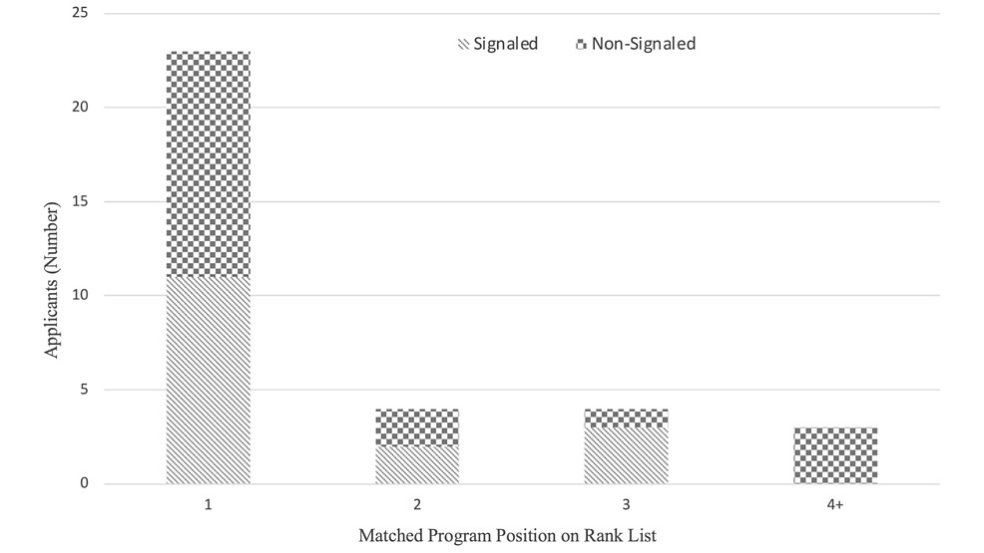
Breakdown of where the applicant matched on their rank list and whether or not that was a signaled program.

All respondents (N=36, 100%) matched to a program on their rank order list and did not have to utilize the Supplemental Offer and Acceptance Program (SOAP). Twenty-four (24, 67%) matched their top choice program, with 33 (92%) matching one of their top three programs. Sixteen (16, 47%, 95% CI 30.64 - 64.14%) matched to a program that they had sent a program signal while 28 (97%, 95% CI 77.69 - 99.56%) matched to a program in a geographic preference division. Match position and associated use of a signal are represented visually in Figure 2.

## Discussion

The program signals and geographic preference divisions in the supplemental application appeared to have a significant positive impact on interview invitations based on data supplied by the applicants. However, their impact on the ultimate results of the Match appeared to be more modest or even negligible.

In the 2022-23 application cycle, IM applicants could send up to seven program signals. Applicants in this study were 2.95 times as likely to receive an interview invitation from a program as they signaled, indicating the utility of this new tool in securing an interview at a highly desired program. Previous studies evaluating the effectiveness of preference signals within and outside of medicine have demonstrated increased odds of receiving an interview invitation if a preference signal is used [11,14]. These data do not, however, describe how program directors directly used or approached these signals. There is some concern that offering too many signals may render each signal less meaningful [13]. However, these findings support the effectiveness of up to seven signals as a valuable tool for applicants in the current virtual application environment.

In the geographic preferences section of the supplemental application, applicants could select up to three geographic divisions that are only visible to programs within the selected region, unless the applicant indicates no geographic preference, which is visible to all programs. Applicants in this study were 1.75 times more likely to receive an interview invitation from a program located within a geographic preference division, indicating that demonstrated geographic interest can increase the likelihood of obtaining an interview invitation. It cannot be concluded that the geographic preference section of the supplemental application is solely responsible for this increased likelihood, however, as there may be other factors that influence an applicant’s probability of receiving an interview invitation within a geographic preference division. For example, the region might encompass the applicant’s home program, hometown, or other connections, which were not accounted for in this study. Studies have shown that applicants are more likely to receive interview invitations from programs within their current geographic area even before the advent of geographic preference signaling; this suggests that these other factors are already being used by programs [15]. Nonetheless, these findings support the effectiveness of giving applicants a platform to express their geographic preferences rather than leaving it to programs to assume.

While indicating geographic preference divisions was effective at increasing the likelihood of an interview invitation, the effect was not as strong as that of program signals. The power of program signals likely lies in their exclusivity; with only seven program signals, participants have the explicit opportunity to indicate a high level of interest in their top programs [13]. In contrast, applicants may select up to three geographic preference divisions, which each contain between three and ten states and many more programs. The smaller effect of geographic preferences on interview invitations, as compared to the effect of program signals, could be explained by a program’s perception of geographic preferences as less specific. With the large number of programs that geographic preferences encompass and the lack of specificity of the geographic preferences signal, they represent a more diluted preference indicator when compared to program signals.

Interestingly, less than half of the participants matched at a program they signaled. This could be due to a multitude of reasons: some programs did not participate in the supplemental application, applicants’ opinions of programs may have changed throughout the interview process, or applicants may have ranked a signaled program highly but not matched there. Additionally, official guidance from the AAMC for the 2022-2023 application cycle recommended that IM applicants not signal their home institution or an institution where they completed an away rotation. Thus, an applicant’s preferences in this cycle may not have been fully captured by program signals. Guidance has shifted for the 2023-2024 cycle; the AAMC now universally recommends that program signaling decisions should only be based on the level of interest in the program [16]. The inclusion of home programs and away rotations in program signals will require further study.

The lack of demonstrable impact on match results should not be interpreted as a failure of program signals. This result is in line with the intention to influence only interview invitations and allows medical students to express their final preferences in their rank list without feeling tied to their original signals.

There are several limitations to these findings. The sample size was relatively small, limiting its statistical power, and only included applicants from North Carolina allopathic medical schools. One osteopathic program in North Carolina was invited to participate but declined. While the approach was intended to minimize selection bias and ensure the inclusion of students at public and private institutions, it may limit the generalizability of the study beyond the state (including non-US MD applicants) or to osteopathic medical schools. Participants’ reported Step 1 and Step 2 scores and match rates were above the national average, so our findings may be more generalizable to an above-average IM applicant. This could also be a reflection of response bias, with students who experienced more favorable outcomes in the match being more likely to complete the survey. There is also the potential for recall bias in answering questions; however, we attempted to minimize this bias by instructing participants to use their ERAS application during the survey. Our findings offer a valuable assessment of the supplemental application from four North Carolina medical schools; however, release of all application data from the AAMC and match data from NRMP would allow for a more complete analysis of the effect of the supplemental application. Preliminary analyses of these data to date, however, have not provided a complete insight into the effect of these changes on the application process as initially described here. Further study with a validated study instrument would be necessary to adequately assess the direct effect of program signals and geographic preferences on match outcomes, as our survey was not adequately powered to evaluate this as a primary outcome. The lack of a validated instrument to complete this study with should be considered in the interpretation of these results.

## Conclusions

The expressed purpose of program signals is to indicate an applicant’s preference at the time of submission of their application. The findings of this study show that for IM applicants from North Carolina schools, that aim was accomplished, with preference signaling significantly increasing the likelihood of an interview invitation. Preference signals should not, however, be used as a proxy to determine whether or not an applicant will rank a program highly. With growing concerns about application inflation, the supplemental application offers applicants powerful tools to assist with securing interview invitations to their top programs and offers programs an additional way to screen applicants.

## Appendices

Below is a copy of the survey sent to potential participants as downloaded from the online survey Software to allow for ease of upload. Display logic is included as well.

Effect of supplemental application on interview invitations and match

Note: It may be helpful to complete this survey with a copy of your ERAS application readily available.

Which medical school in North Carolina do you attend?

o Brody School of Medicine at East Carolina University (1)

o Duke University School of Medicine (3)

o University of North Carolina School of Medicine (4)

o Wake Forest School of Medicine (5)

Did you apply into categorical Internal Medicine (i.e. not only preliminary) during the ERAS 2022-2023 application cycle?

o Yes (1) 

o No (2) 

Skip To: End of Survey If Did you apply into categorical Internal Medicine (i.e. not only preliminary) during the ERAS 2022... = No

Did you apply into any other specialties?

o Yes (1) 

o No (2)

Display this question

If Did you apply into any other specialties?  = Yes

Which additional specialty or specialties did you apply into?

▢        Anesthesiology (1)

▢        Cardiothoracic Surgery (2)

▢        Dermatology (3)

▢        Diagnostic Radiology (4)

▢        Emergency Medicine (5)

▢        Family Medicine (6)

▢        Med Peds (7)

▢        Neurosurgery (8)

▢        Neurology (9)

▢        OBGyn (10)

▢        Ophthalmology (11)

▢        Orthopedic Surgery (12)

▢        Otolaryngology (13)

▢        Pediatrics (14)

▢        PM&R (15)

▢        Plastic Surgery (16)

▢        Preventative Medicine (17)

▢        Psychiatry (18)

▢        Radiation Oncology (19)

▢        Surgery - General (20)

▢        Urology (21)

▢        Vascular Surgery (22)

▢        Other (23) __________________________________________________

Display this question

If Did you apply into any other specialties?  = Yes

Was Internal Medicine your first choice for specialty?

o Yes (1)

o No (2)

Did you participate in the Couples Match?

o Yes (1)

o No (2)

How many Internal Medicine programs did you apply to? 

_______________________________________________________________

Did you withdraw from any programs before receiving an interview invitation? 

o Yes (2)

o No (1)

Display this question

Did you withdraw from any programs before receiving an interview invitation?  = Yes

How many programs did you withdraw from before receiving an interview invitation?

_______________________________________________________________

Please select the number of publications, work experiences, volunteer experiences, and research experiences on your ERAS application.

0 (1)

1 (2)

2 (3)

3 (4)

4 (5)

5 (6)

6 (7)

7 (8)

8 (9)

9 (10)

10+ (11)

Publications (1)

Work Experiences (2)

Volunteer Experiences (3

Research Experiences (4)

Did you participate in the Internal Medicine Supplemental ERAS application?

o Yes (2)

o No (1)

Display this question

If Did you participate in the Internal Medicine Supplemental ERAS application?  = Yes

How many meaningful experiences did you list?

o 0 (1)

o 1 (2)

o 2 (3)

o 3 (4)

o 4 (5)

o 5 (6)

Display this question

If Did you participate in the Internal Medicine Supplemental ERAS application?  = Yes

Did you complete the "Past Experiences: Other Impactful Experiences" section of the supplemental application?

o Yes (2)

o No (1)

Display this question

If Did you participate in the Internal Medicine Supplemental ERAS application?  = Yes

How many geographic preference regions did you select?

o 0 (1)

o 1 (2)

o 2 (3)

o 3 (4)

Display this question

If How many geographic preference regions did you select?  != 0

Of the programs you applied to, how many were in a geographic preference region?

________________________________________________________________

Display this question

If How many geographic preference regions did you select?  != 0

For reference, the geographic divisions are as follows: - Pacific: AK, CA, HI, OR, WA - Mountain: AZ, CO, ID, MT, NM, NV, UT, WY - West North Central: IA, KS, MN, MO, NE, ND, SD - East North Central: IL, IN, MI, OH, WI - West South Central: AR, LA, OK, TX - East South Central: AL, KY, MS, TN - South Atlantic: DC, DE, FL, GA, MD, NC, PR, SC, VA, WV - Middle Atlantic: NJ, NY, PA - New England: CT, ME, MA, NH, RI, VT

Display this question

If Did you participate in the Internal Medicine Supplemental ERAS application?  = Yes

What did you select for "Geographic Preferences: Setting Preferences"?

o Strong preference for RURAL (1)

o Slight preference for RURAL (2)

o No preference (3)

o Slight preference for URBAN (4)

o Strong preference for URBAN (5)

Display this question

If Did you participate in the Internal Medicine Supplemental ERAS application?  = Yes

How many programs did you signal?

o 0 (1)

o 1 (2)

o 2 (3)

o 3 (4)

o 4 (5)

o 5 (6)

o 6 (7)

o 7 (8)

How many interview invitations did you receive?

________________________________________________________________

Display this question

If Did you participate in the Internal Medicine Supplemental ERAS application?  = Yes

Of the programs that you signaled, how many offered you an interview?

________________________________________________________________

Display this question

If Did you participate in the Internal Medicine Supplemental ERAS application?  = Yes

Of the signaled programs who offered you an interview, how many were also in your geographic preference area?

________________________________________________________________

Display this question

If Did you participate in the Internal Medicine Supplemental ERAS application?  = Yes

For reference, the geographic divisions are as follows - Pacific: AK, CA, HI, OR, WA - Mountain: AZ, CO, ID, MT, NM, NV, UT, WY - West North Central: IA, KS, MN, MO, NE, ND, SD - East North Central: IL, IN, MI, OH, WI - West South Central: AR, LA, OK, TX - East South Central: AL, KY, MS, TN - South Atlantic: DC, DE, FL, GA, MD, NC, PR, SC, VA, WV - Middle Atlantic: NJ, NY, PA - New England: CT, ME, MA, NH, RI, VT

Display this question

If Did you participate in the Internal Medicine Supplemental ERAS application?  = Yes

Of the programs you did not signal, how many offered you an interview and were also in your geographic preference area?

________________________________________________________________

Did you cancel any interviews? 

o Yes (1)

o No (2)

Display this question

If Did you cancel any interviews?  = Yes

How many interviews did you cancel

________________________________________________________________

How many letters of interest, if any, did you send? If none, enter "0".

________________________________________________________________

Display this question

If If How many letters of interest, if any, did you send? If none, enter "0". Text Response Is Greater Than  0

How many interview invitations came from programs after you sent a letter of interest?

________________________________________________________________

Did you send thank you notes after interviews?

o Yes (3)

o Sometimes (2)

o No (1)

Did you send a letter of intent to the program you put at the top of your rank list?

o Yes (1)

o No (2)

How many programs did you rank?

________________________________________________________________

Did you match to a program on your rank list?

o Yes (2)

o No (1)

Display this question

If Did you match to a program on your rank list? = No

Did you match via the SOAP process?

o Yes (2)

o No (1)

Display this question

If Did you match to a program on your rank list? = Yes

Where did you match on your rank list? (please write as 1, 2, 3, etc.)

________________________________________________________________

Display this question

If How many programs did you signal?  != 0

And Did you participate in the Internal Medicine Supplemental ERAS application?  = Yes

Did you match at a program you had signaled?

o Yes (2)

o No (1)

Display this question

If How many geographic preference regions did you select?  != 0

And Did you participate in the Internal Medicine Supplemental ERAS application?  = Yes

Did you match at a program in one of your geographic preference regions?

o Yes (1)

o No (2)

Which gender do you most identify with?

o Cis-Male (1)

o Cis-Female (2)

o Trans-Male (3)

o Trans-Female (4)

o Non-binary / third gender (5)

o Prefer not to say (6)

Please select your age

o < 26 (1)

o 26-30 (2)

o 31-35 (3)

o 36-40 (4)

o 41-45 (5)

o 46-50 (6)

o > 50 (7)

Which race or ethnicity best describes you? (select all that apply)

▢        American Indian or Alaskan Native (1)

▢        Asian/Pacific Islander (2)

▢        Black or African American (3)

▢        Hispanic (4)

▢        White/Caucasian (5)

▢        Multiple Ethnicity/Other (Please Specify)  (6) __________________________________________________

Did you take Step 1 pass/fail?

o Yes (1)

o No (2)

Display this question

If Did you take Step 1 pass/fail? = No

What was your three digit Step 1 score on your first attempt? 

________________________________________________________________

Display this question

If Did you take Step 1 pass/fail? = Yes

Did you pass or fail Step 1 on your first attempt?

o Pass (1)

o Fail (2)

What was your three digit Step 2 score on your first attempt?

________________________________________________________________

Are you a member of AOA?

o Yes (2)

o No (1)

o No - No Chapter at my School (3)

Are you a member of the Gold Humanism Society?

o Yes (2)

o No (1)

o No - No Chapter at my School (3)
